# Degradation of cyclin B is critical for nuclear division in *Trypanosoma brucei*

**DOI:** 10.1242/bio.031609

**Published:** 2018-03-12

**Authors:** Hanako Hayashi, Bungo Akiyoshi

**Affiliations:** Department of Biochemistry, University of Oxford, Oxford, OX1 3QU, UK

**Keywords:** Cell cycle, Spindle checkpoint, Kinetoplastid, *Trypanosoma brucei*, Kinetochore, Cyclin B

## Abstract

Kinetoplastids have a nucleus that contains the nuclear genome and a kinetoplast that contains the mitochondrial genome. These single-copy organelles must be duplicated and segregated faithfully to daughter cells at each cell division. In *Trypanosoma brucei*, although duplication of both organelles starts around the same time, segregation of the kinetoplast precedes that of the nucleus. Cytokinesis subsequently takes place so that daughter cells inherit a single copy of each organelle. Very little is known about the molecular mechanism that governs the timing of these events. Furthermore, it is thought that *T. brucei* lacks a spindle checkpoint that delays the onset of nuclear division in response to spindle defects. Here we show that a mitotic cyclin CYC6 has a dynamic localization pattern during the cell cycle, including kinetochore localization. Using CYC6 as a molecular cell cycle marker, we confirmed that *T. brucei* cannot delay the onset of anaphase in response to a bipolar spindle assembly defect. Interestingly, expression of a stabilized form of CYC6 caused the nucleus to arrest in a metaphase-like state without preventing cytokinesis. We propose that trypanosomes have an ability to regulate the timing of nuclear division by modulating the CYC6 protein level, without a spindle checkpoint.

## Abstract (precis)

**Summary:** Trypanosomes do not have a spindle checkpoint but have an ability to regulate the timing of nuclear division by modulating the cyclin B protein level.

## INTRODUCTION

Accurate transmission of genetic material to offspring is essential for the survival of organisms. The genome in eukaryotes exists in different organelles such as the nucleus, mitochondria, and plastids. Nuclear DNA is duplicated during S phase and segregated equally to daughter cells during M phase. Kinetochores are the macromolecular protein complexes that assemble onto centromeric DNA and interact with spindle microtubules. It is essential that sister kinetochores attach to spindle microtubules emanating from opposite poles in metaphase so that sister chromatids segregate away from each other during anaphase. Cells possess a surveillance mechanism, called the spindle checkpoint, that delays the onset of anaphase in response to defects in kinetochore-microtubule attachments (London and Biggins, 2014; Musacchio, 2015). Once all sister kinetochores have achieved proper bi-oriented attachments, the spindle checkpoint is satisfied. This results in the ubiquitylation of two key targets, cyclin B and securin, by the anaphase-promoting complex (APC/C), leading to their destruction by proteasomes.

**Fig. 1.**
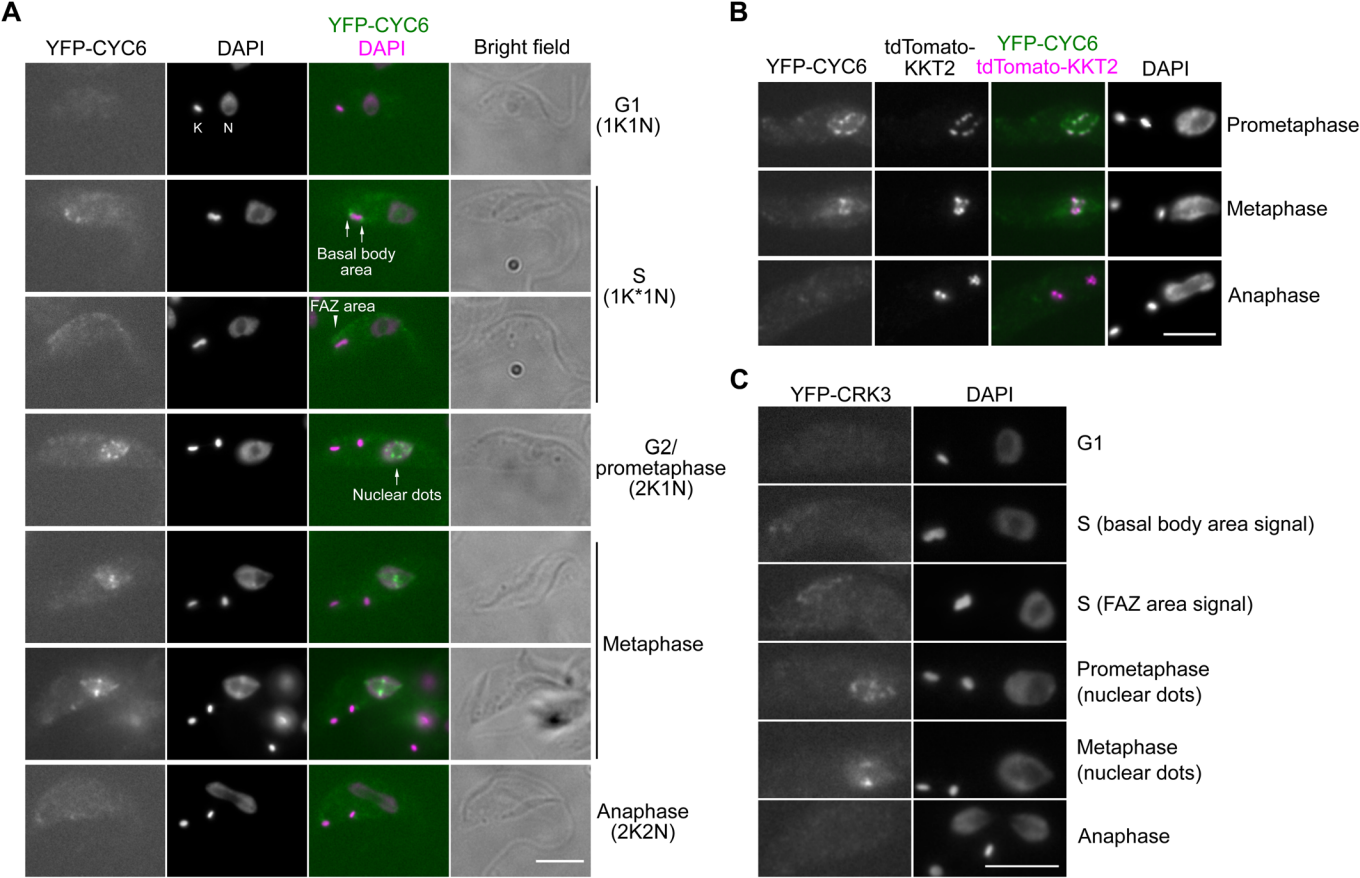
**Cyclin B^CYC6^ is enriched at kinetochores in metaphase and disappears in anaphase.** (A) CYC6 has a dynamic localization pattern during the cell cycle. Examples of procyclic form cells that express YFP-CYC6 are shown (cell line BAP426). K and N stands for the kinetoplast and nucleus, respectively. (B) CYC6 nuclear dots partially co-localize with a kinetochore protein, KKT2 (BAP1005). (C) CRK3 has nuclear dots in metaphase and disappears in anaphase (BAP463). Scale bars: 5 µm.

In contrast to nuclear DNA, the mechanism of mitochondrial DNA transmission varies among eukaryotes. For example, in animals that have a high copy number of mitochondria, transmission of mitochondrial DNA is thought to occur randomly (Westermann, 2010). On the other hand, a single mitochondrion is present in many unicellular eukaryotes, such as kinetoplastids, *Plasmodium falciparum*, and *Cyanidioschyzon merolae* (Robinson and Gull, 1991; Itoh et al., 1997; Okamoto et al., 2009). The timing of duplication and partition of their mitochondria must be coordinated with the cell cycle machinery in these organisms. Kinetoplastids are a group of unicellular organisms that are characterized by the unique structure called the kinetoplast, which is a network of multiple copies of mitochondrial DNA (termed the kDNA) enclosed in a single mitochondrion (Vickerman, 1962). They are evolutionarily divergent from commonly studied model eukaryotes (e.g. yeasts, worms, flies, and humans) (Cavalier-Smith, 2010; Walker et al., 2011), so understanding their biology can provide insights into the extent of conservation or divergence in eukaryotes. Among various kinetoplastids studied thus far, the mechanism of cell cycle is best characterized in *Trypanosoma brucei*, the causative agent of human African trypanosomiasis (for reviews, see McKean, 2003; Hammarton, 2007; Vaughan and Gull, 2008; Li, 2012). *T. brucei* has a canonical cell cycle for nuclear events (G1, S, G2, and M phases). G1 cells have a single kinetoplast and nucleus (termed 1K1N) (see Fig. 1A). Duplication of kinetoplast DNA starts almost simultaneously with that of nuclear DNA, but completes earlier (Woodward and Gull, 1990; Siegel et al., 2008). Segregation of kDNA depends on that of basal bodies and occurs during the nuclear S phase, creating 2K1N cells (Robinson and Gull, 1991; Ogbadoyi et al., 2003; Davidge et al., 2006). Trypanosomes do not break down their nuclear envelope (closed mitosis), and an intranuclear mitotic spindle is assembled in the nucleus during M phase (Vickerman and Preston, 1970; Ogbadoyi et al., 2000). Sister kinetochores align at the metaphase plate during metaphase, followed by the separation of nuclear DNA in anaphase (creating 2K2N cells) and the split of cells by cytokinesis (Sherwin and Gull, 1989; Woodward and Gull, 1990). It is essential that replication and segregation of these organelles occur prior to cytokinesis in a coordinated manner so that daughter cells inherit a copy of each. Little is known about the underlying molecular mechanism.

Available evidence suggests that *T. brucei* is not capable of halting their cell cycle in response to various defects in the nucleus. For example, when bipolar spindle assembly is blocked in procyclic (insect form) cells, they undergo cytokinesis without a noticeable delay despite a lack of nuclear division (Robinson et al., 1995; Ploubidou et al., 1999). This results in the formation of one daughter cell that has one kinetoplast and no nucleus (1K0N, termed zoid) and another cell that has one kinetoplast and a nucleus of tetraploid DNA content, suggesting that the spindle checkpoint is not operational (Ploubidou et al., 1999). In fact, most of the spindle checkpoint components (i.e. Mps1, Mad1, Mad3/BubR1, Bub1, Bub3) are not found in *T. brucei* or other kinetoplastids. Although a Mad2 homolog is present, this protein localizes at basal bodies, not kinetochores (Akiyoshi and Gull, 2013). It is therefore thought that trypanosomes cannot delay cytokinesis even when nuclear division fails to occur. Yet, there must be a mechanism to coordinate the segregation of nuclear DNA with cytokinesis in unperturbed cells. One possibility is the presence of a cell cycle oscillator that triggers cell cycle events in a defined sequence even without feedback control systems. The best characterized components of cell cycle oscillators are cyclin/CDK (cyclin-dependent kinase) complexes (Nurse, 1990; Morgan, 1997; Gérard et al., 2015). The rise and fall of their kinase activities trigger cell cycle events in a set sequence. For example, increased activities of mitotic CDK complexes promote entry into M phase and various mitotic events, whereas their decrease is essential for exit from mitosis. *T. brucei* has 10 cyclins and 11 CDKs, among which CYC6/CRK3 is the major mitotic cyclin/CDK complex in *T. brucei* (CYC6 is also known as CycB2) (Li and Wang, 2003; Hammarton et al., 2003). When degradation of CYC6 was inhibited by proteasome inhibitors or APC/C downregulation, cells accumulated in a metaphase-like state with a bipolar spindle (Mutomba et al., 1997; Kumar and Wang, 2005). These observations suggested that degradation of cyclin B could be a trigger for the metaphase-anaphase transition. Here we directly tested this possibility by expressing a non-degradable version of CYC6 in *T. brucei*.

## RESULTS

### Identification of cyclin B^CYC6^ as a molecular cell cycle marker

Cellular localization of CYC6 has not been reported thus far, so we first examined it by endogenously tagging CYC6 with an N-terminal YFP tag in *T. brucei* procyclic cells. We observed the following localization pattern (Fig. 1A). There was no distinct signal in G1 cells. From S phase onwards, CYC6 was found near the basal body area and the flagellum attachment zone (FAZ). From G2 to metaphase, nuclear signal was observed with significant enrichment at kinetochore regions in metaphase. In fact, these nuclear dots co-localized with a kinetochore marker protein, KKT2 (Fig. 1B). CYC6 disappeared from the nucleus in anaphase. We obtained similar results for CRK3, which formed nuclear dots in metaphase and disappeared in anaphase (Fig. 1C). Thus, CYC6 and CRK3 exhibit a dynamic localization pattern depending on cell cycle stages, and these proteins can therefore be used as a molecular cell cycle marker.

### Cyclin B^CYC6^ is important for bipolar spindle assembly

**Fig. 2.**
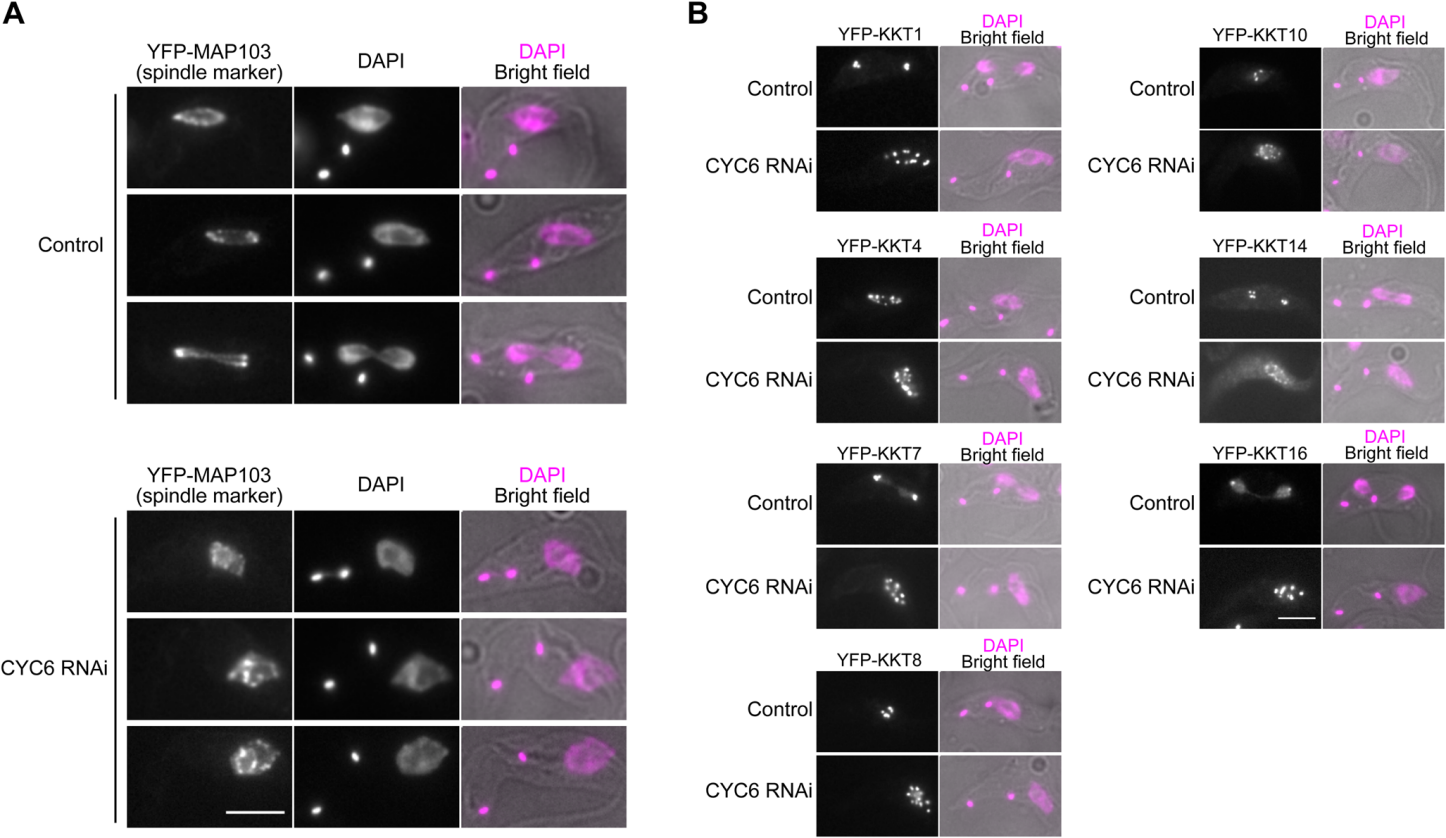
**Cyclin B^CYC6^ is important for bipolar spindle assembly, but dispensable for the localization of many kinetochore proteins.** (A) Bipolar spindle formation was perturbed upon induction of CYC6 RNAi. Cells expressing YFP-MAP103 (a marker for spindle microtubules) were fixed at 24 h post-induction (BAP504). (B) Kinetochore localization of KKT1, KKT4, KKT7, KKT8, KKT10, KKT14, and KKT16 proteins was not affected by CYC6 depletion (BAP503, BAP585, BAP505, BAP593, BAP596, BAP506, and BAP604, respectively). Examples of 2K1N (prometaphase/metaphase) or 2K2N (anaphase) cells expressing indicated YFP-KKT proteins fixed at 24 h post-induction are shown. Note that CYC6-depleted cells have spindle assembly defects, and their chromosomes fail to align onto the metaphase plate. Scale bars: 5 µm.

CDK activities are known to be important for kinetochore assembly in some eukaryotes, including humans (Gascoigne and Cheeseman, 2013). The finding that CYC6 localizes at kinetochores from G2 to metaphase in trypanosomes prompted us to study its importance for kinetochore assembly. We therefore depleted CYC6 by RNAi-mediated knockdown (Ngô et al., 1998). We confirmed that CYC6 is essential for cell growth (Fig. S1), as previously reported (Li and Wang, 2003; Hammarton et al., 2003). Because cyclin/CDK activities are known to be important for various mitotic events (Bishop et al., 2000), we first examined bipolar spindle formation. We used a spindle marker protein that we identified from our previous tagging screen (ORF Tb927.11.14370) (Archer et al., 2011; Akiyoshi and Gull, 2014). This protein had a localization pattern characteristic of spindle microtubules, so we named it MAP103 for microtubule-associated protein 103 kDa (Fig. S2). We observed defective spindle microtubules in CYC6-depleted cells, suggesting that CDK activities are essential for proper bipolar spindle assembly (Fig. 2A). Under these conditions, however, nuclear dot formation of all KKT proteins we examined was not affected (KKT1, KKT4, KKT7, KKT8, KKT10, KKT14, KKT16) (Fig. 2B). Therefore, CYC6 is dispensable for the localization of these kinetochore proteins in procyclic cells. However, we currently do not know whether kinetochores are assembled properly in CYC6-depleted cells.

### Cells fail to delay the onset of anaphase in response to spindle defects

**Fig. 3.**
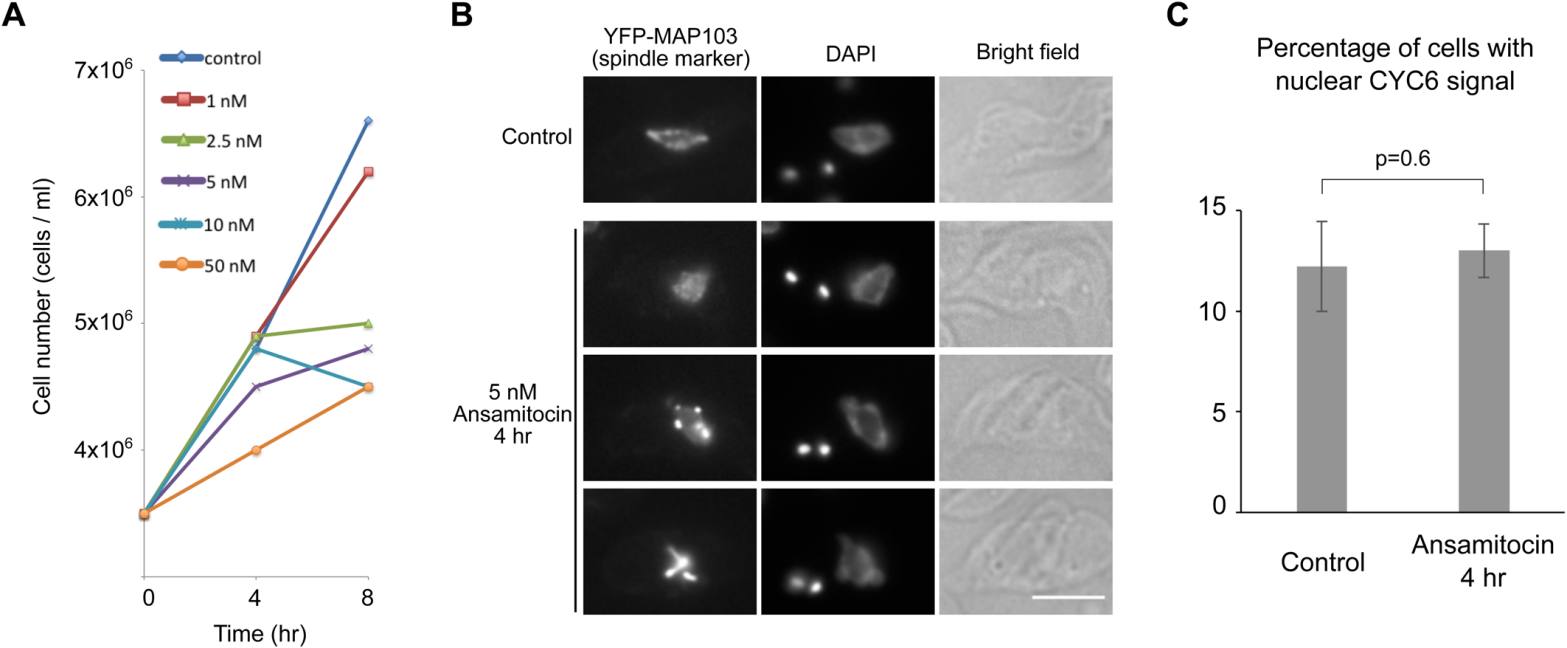
**Spindle assembly defects do not prevent cyclin B^CYC6^ degradation in the nucleus.** (A) Growth curves of control and ansamitocin-treated cultures show a concentration-dependent growth inhibition (BAP125). (B) Ansamitocin prevents bipolar spindle assembly. Cells expressing YFP-MAP103 (BAP79) were treated with 5 nM ansamitocin for 4 h and fixed. Scale bar: 5 µm. (C) Ansamitocin treatment does not result in the accumulation of nuclear CYC6-positive cells. Cells expressing YFP-CYC6 (BAP426) were treated with 5 nM ansamitocin for 4 h and fixed. Three hundred cells were counted for each sample, and experiments were performed three times. Error bars represent standard deviation. *P*-value was obtained by two-tailed, unpaired *t*-test.

We next used CYC6 as a molecular cell cycle marker to examine the effect of drugs. We first used an anti-microtubule agent, ansamitocin, to test whether bipolar spindle assembly defects affect cell cycle progression (Robinson and Gull, 1991). By testing various concentrations of ansamitocin, we found that 5 nM of ansamitocin immediately slowed down cell growth (Fig. 3A). After a 4-h treatment, nuclear division and bipolar spindle assembly were perturbed as expected (Fig. 3B). In this condition, however, we found no significant enrichment of nuclear CYC6-positive cells (Fig. 3C). This corroborates previous studies (Ploubidou et al., 1999) and confirms that trypanosomes are not capable of delaying the onset of anaphase in response to spindle damage.

### Stabilization of cyclin B^CYC6^ causes metaphase arrest in the nucleus

**Fig. 4.**
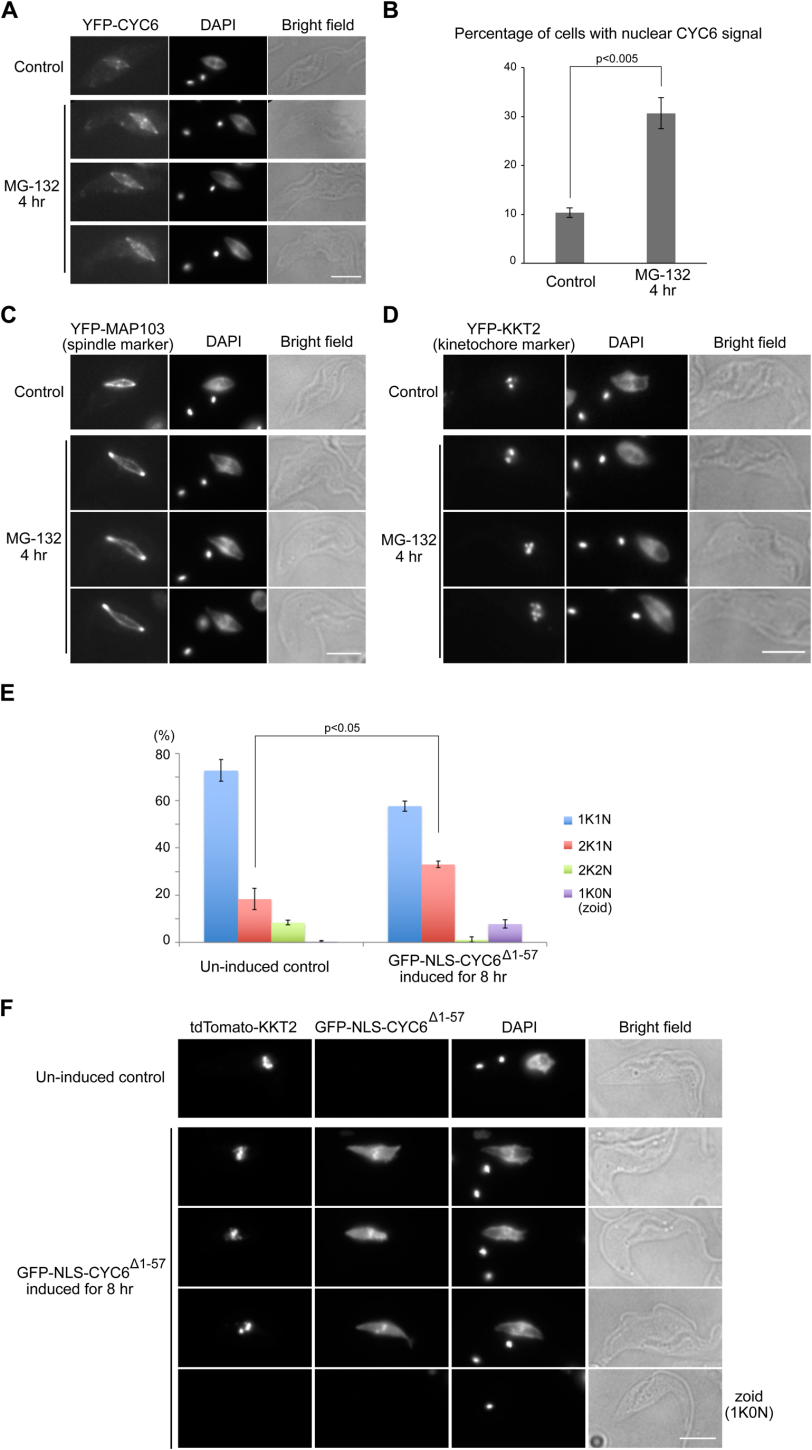
**Non-degradable cyclin B^CYC6^ prevents nuclear division.** (A–D) MG-132 treatment causes metaphase arrest. Cells expressing YFP-CYC6 (A,B: BAP426), YFP-MAP103 (C: BAP79), or YFP-KKT2 (D: BAP122) were treated with 10 µM MG-132 for 4 h and fixed, showing that a higher ratio of cells have nuclear CYC6 signal with a bipolar spindle and aligned kinetochores upon MG-132 treatment. For quantification of nuclear CYC6-positive cells (B), 300 cells were counted for each sample, and experiments were performed three times. (E,F) Expression of a non-degradable CYC6 protein in the nucleus delays nuclear division. GFP-NLS-CYC6^Δ1–57^ expression was induced with 0.1 µg ml^−1^ doxycycline in cells that have tdTomato-KKT2 (BAP945) for 8 h. Four hundred cells were counted for each condition, and experiments were performed three times. Error bars represent standard deviation. *P*-values were obtained by two-tailed, unpaired *t*-test. Scale bars: 5 µm.

We next examined the effect of cyclin B stabilization for cell cycle progression. We first used a proteasome inhibitor MG-132 that blocked cell cycle progression and stabilized the CYC6 protein (Mutomba et al., 1997; Bessat et al., 2013). When cells expressing YFP-CYC6 were treated with 10 µM MG-132 for 4 h, ∼30% of cells had nuclear CYC6 signal (compared to ∼10% in control), suggesting that the nucleus arrested prior to anaphase (Fig. 4A,B). Indeed, these cells had a bipolar spindle (often elongated) and most of their kinetochores were aligned at the metaphase plate (Fig. 4C,D). We also noted that the distance between the two kinetoplast DNA in these cells was often greater than that in control metaphase cells. These results suggest that, upon MG-132 treatment, trypanosomes arrest the nucleus in a metaphase-like state in which cyclin B is not degraded, although their cytoplasm transits to an anaphase-like state.

Because MG-132 treatment affects the protein level of many proteins, we next tested whether the presence of cyclin B in the nucleus is sufficient to prevent nuclear division. Overexpression of wild-type CYC6 did not affect cell growth (data not shown). In other eukaryotes, cyclin B has degradation motifs in the N-terminal region and its deletion makes the protein insensitive to degradation (Glotzer et al., 1991; Surana et al., 1993; Yamano et al., 1996; Chang et al., 2003). Similarly, a putative destruction box (D-box) was found in *T. brucei* CYC6 (Fig. S3, residues 54-57). We therefore made a putative non-degradable form of CYC6 fused with a nuclear localization signal (GFP-NLS-CYC6^Δ1–57^). Interestingly, we detected a decrease in 2K2N cells and significant accumulation of 2K1N cells upon expression of the non-degradable CYC6 for 8 h (Fig. 4E), suggesting that the nucleus was arrested in a metaphase-like state. Indeed, kinetochores were aligned at the metaphase plate in these cells (Fig. 4F). We also detected an increase in the number of zoids (1K0N cells). This implies that cytokinesis occurred despite the lack of nuclear division (Fig. 4E,F). These results show that GFP-NLS-CYC6^Δ1–57^ is capable of arresting the nucleus in a metaphase-like state, although it cannot stop cytokinesis. Taken together, our data show that trypanosomes have an ability to control the timing of nuclear division by modulating the degradation of a mitotic cyclin in the nucleus.

## DISCUSSION

Previous studies observed the formation of zoids despite a lack of nuclear division due to spindle damage (Ploubidou et al., 1999), cyclin/CDK depletion (Hammarton et al., 2003; Li and Wang, 2003; Tu and Wang, 2004), or expression of a non-degradable cohesin subunit SCC1 (Gluenz et al., 2008). These studies strongly suggested that *T. brucei* cannot prevent cytokinesis in response to a lack of nuclear division at least in procyclic cells [although this is likely to be the case in bloodstream form cells too: see (Gluenz et al., 2008)]. In this study, we established CYC6 as a molecular marker for cell cycle progression, and confirmed that trypanosomes indeed failed to delay the anaphase onset in response to spindle damage. This implies that the timing mechanism of the nuclear cell cycle progression is likely governed by an intrinsic cell cycle timer, as observed in embryonic divisions (Yang and Ferrell, 2013; Yuan and O'Farrell, 2015) and in spindle checkpoint mutants of yeasts, flies, and human HAP1 cells (Hoyt et al., 1991; Li and Murray, 1991; Buffin et al., 2007; Raaijmakers et al., 2018).

Interestingly, we found that expression of non-degradable cyclin B can delay the onset of anaphase. This means that trypanosomes could potentially coordinate the timing of nuclear division with that of cytokinesis by regulating the CYC6 protein level in the nucleus. Because APC/C is responsible for the degradation of mitotic cyclins, understanding its regulatory mechanism is of critical importance. It is interesting to note that two kinetochore proteins (KKT4 and KKT20) co-purified with several components of the APC/C (Akiyoshi and Gull, 2014; Nerusheva and Akiyoshi, 2016), suggesting that kinetochores may directly regulate APC/C activities. It will be important to understand the underlying mechanism.

It remains unclear how the timing of cytokinesis onset is determined in trypanosomes. It has been suggested that it may be the segregation of basal bodies, rather than that of the nucleus, that is linked to cytokinesis in trypanosomes (Ploubidou et al., 1999). Interestingly, CYC6 signal was found not only at kinetochores but also near the basal body area and the flagellum attachment zone. Therefore, CYC6 might also have an ability to regulate the onset of cytokinesis, which will need to be tested in future studies.

## MATERIALS AND METHODS

### Trypanosome cells

All trypanosome cell lines used in this study were derived from *T. brucei* SmOxP927 procyclic form cells (TREU 927/4 expressing T7 RNA polymerase and the tetracycline repressor to allow inducible expression) (Poon et al., 2012) and are listed in Table S1. Cells were grown at 28°C in SDM-79 medium supplemented with 10% (v/v) heat-inactivated fetal calf serum (Brun and Schönenberger, 1979). Cell growth was monitored using a CASY cell counter and analyzer system (Roche, Basel, Switzerland). RNAi was induced with doxycycline at a final concentration of 1 µg ml^−1^. Non-degradable CYC6 was expressed with doxycycline at 0.1 µg ml^−1^. Ansamitocin P-3 was purchased from Abcam (catalog number, ab144546) and MG-132 was purchased from Merck (catalog number, 474790, Darmstadt, Germany). We used sample sizes commonly used in the field. All experiments were performed at least twice unless otherwise noted.

### Tagging, cloning, transfections, and microscopy

Plasmids and primers used in this study are listed in Tables S2 and S3, respectively. Endogenous tdTomato tagging was performed using pBA148 (Akiyoshi and Gull, 2014). YFP tagging was performed using pEnT5-Y (for KKTs and MAP103) or pBA106 (for CYC6 and CRK3) tagging vectors. pBA106 is a modified version of the pEnT5-Y vector (Kelly et al., 2007) to allow N-terminal 3FLAG-6HIS-YFP tagging. A targeting sequence for the CRK3 tagging (consisting of *Xba*I site, 4–250 bp of the CRK3 coding sequence, *Not*I site, 250 bp of CRK3 5′UTR, *Bam*HI site) was synthesized by GeneArt (Thermo Fisher Scientific). To make pBA106, a synthetic DNA fragment that encodes a 3FLAG-6HIS tag (made by annealing BA403 and BA404) was ligated into pEnT5-Y using *Hind*III and *Spe*I sites. For generation of the inducible CYC6 RNAi cell line, a 424 bp fragment targeting 378–801 bp of the CYC6 coding sequence was amplified from genomic DNA and cloned into the p2T7-177 vector (Wickstead et al., 2002), creating pBA734. To make a non-degradable version of CYC6 with an N-terminal GFP-NLS tag (pBA1319: GFP-NLS-CYC6^Δ1–57^), a DNA fragment encoding CYC6^58–426^ was amplified from genomic DNA and cloned into pBA310 (Nerusheva and Akiyoshi, 2016) using *Pac*I and *Asc*I sites. Plasmids linearized by *Not*I were transfected to trypanosomes by electroporation into an endogenous locus (pEnT5-Y, pBA106, and pBA148 derivatives) or 177 bp repeats on minichromosomes (p2T7-177 and pBA310 derivatives). Transfected cells were selected by the addition of 25 µg ml^−1^ hygromycin (pEnT5-Y and pBA106 derivatives), 10 µg ml^−1^ blasticidin (pBA148 derivatives), or 5 µg ml^−1^ phleomycin (p2T7-177 and pBA310 derivatives). Microscopy was performed essentially as previously described using a Leica DM5500 B microscope (Leica Microsystems, Wetzlar, Germany) housed in the Keith Gull's laboratory (Akiyoshi and Gull, 2014) to image YFP-MAP103 or DeltaVision fluorescence microscope (Applied Precision/GE Healthcare, Amersham, UK) housed in the Micron Oxford Advanced Bioimaging Unit (Nerusheva and Akiyoshi, 2016) for all other experiments.

## Supplementary Material

Supplementary information
