# Supplementary material for: Degradation of cyclin B is critical for nuclear division in Trypanosoma brucei
Source: Biol Open. 2018 Mar 12;7(3):bio031609. doi: 10.1242/bio.031609 (PMC5898267; doi:10.1242/bio.031609)
Supplement: Supplementary information [file biolopen-7-031609-s1.pdf]

Supplemental materials for

Degradation of cyclin B is critical for nuclear division in *Trypanosoma brucei*

Hanako Hayashi and Bungo Akiyoshi

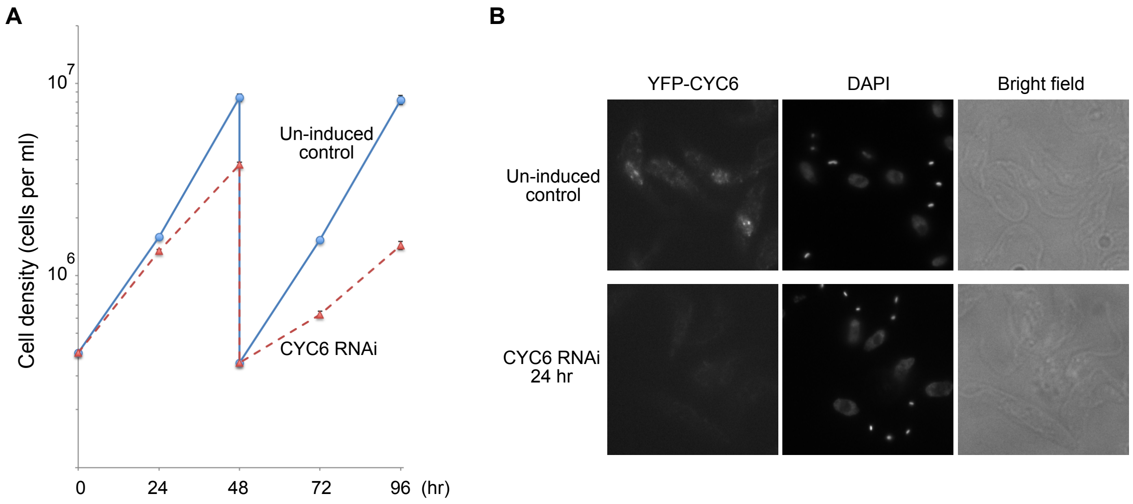

Figure S1. CYC6 is essential for cell growth

(A) RNAi-mediated knockdown of CYC6 causes a severe growth defect (cell line BAP507), as reported previously (Hammarton et al., 2003; Li and Wang, 2003). The experiment was performed in triplicates, and the mean value is shown. Error bars represent standard deviation.

(B) YFP-CYC6 signal is reduced upon induction of CYC6 RNAi for 24 hours. Examples of cells expressing YFP-CYC6 without (top) or with (bottom) CYC6 RNAi induction are shown.

Biology Open • Supplementary information

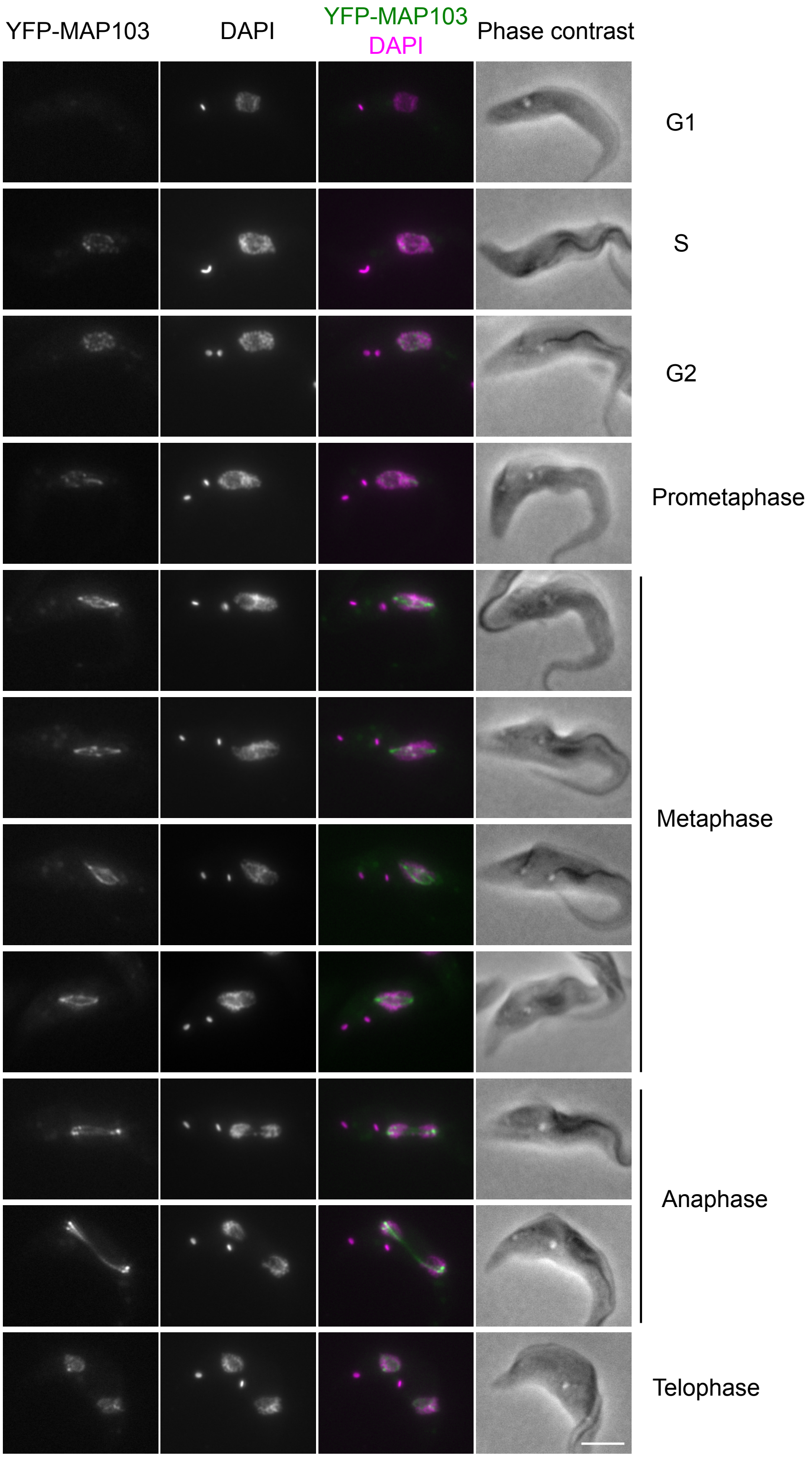

Figure S2. MAP103 has a localization pattern reminiscent of spindle microtubules during mitosis

Examples of cells expressing YFP-MAP103 at indicated cell cycle stages are shown (cell line BAP79). Bar, 5 µm.

Biology Open • Supplementary information

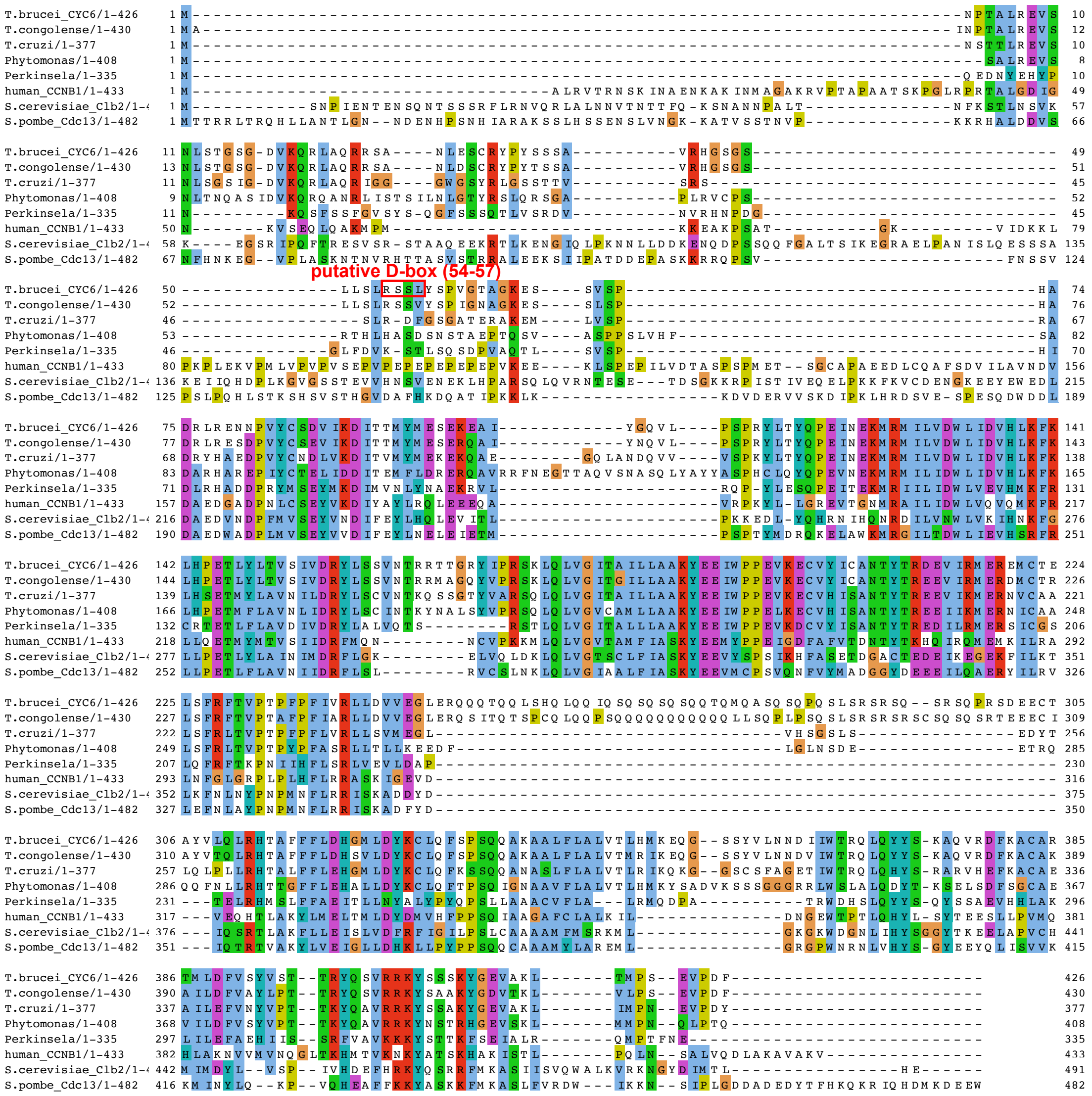

Figure S3. Multiple sequence alignment of cyclin B

Protein sequences of cyclin B from kinetoplastids (*T. brucei, T. congolense, T. cruzi, Phytomonas,* and *Perkinsela*) and other eukaryotes (human, *S. cerevisiae,* and *S. pombe*) were aligned using MAFFT (L-INS-i) (Katoh and Standley, 2013) and visualized with a CLUSTALX coloring scheme in Jalview (Waterhouse et al., 2009). A putative D-box motif in *T. brucei* CYC6 is highlighted in a red box.

Table S1. Trypanosome cell lines used in this study.

Strain	Description
SmOxP9	Parental cell line that expresses TetR and T7 RNAP (Kelly et al., 2007)
BAP79	TY-YFP-MAP103 (this study)
BAP122	TY-YFP-KKT2 (Akiyoshi and Gull, 2014)
BAP125	TY-YFP-KKT4 (Akiyoshi and Gull, 2014)
BAP426	3FLAG-6HIS-YFP-CYC6 (this study)
BAP463	3FLAG-6HIS-YFP-CRK3 (this study)
BAP503	TY-YFP-KKT1, Inducible CYC6 RNAi (this study)
BAP504	TY-YFP-MAP103, Inducible CYC6 RNAi (this study)
BAP505	TY-YFP-KKT7, Inducible CYC6 RNAi (this study)
BAP506	TY-YFP-KKT14, Inducible CYC6 RNAi (this study)
BAP507	3FLAG-6HIS-YFP-CYC6, Inducible CYC6 RNAi (this study)
BAP585	TY-YFP-KKT4, Inducible CYC6 RNAi (this study)
BAP593	TY-YFP-KKT8, Inducible CYC6 RNAi (this study)
BAP596	TY-YFP-KKT10, Inducible CYC6 RNAi (this study)
BAP604	TY-YFP-KKT16, Inducible CYC6 RNAi (this study)
BAP945	TY-tdTomato-KKT2, Inducible GFP-NLS-CYC6$^{\Delta 1-57}$ (this study)
BAP1005	3FLAG-6HIS-YFP-CYC6, TY-tdTomato-KKT2 (this study)

Table S2. Plasmids used in this study.

Name	Descriptions
pEnT5-Y	TY-YFP tagging vector, Hygromycin (Kelly et al., 2007)
p2T7-177	Inducible RNAi vector, integrate at 177 bp repeats (Wickstead et al., 2002)
pBA18	TY-YFP-KKT1 tagging vector, Hygromycin (Akiyoshi and Gull, 2014)
pBA31	TY-YFP-MAP103 tagging vector, Hygromycin (this study)
pBA67	TY-YFP-KKT2 tagging vector, Hygromycin (Akiyoshi and Gull, 2014)
pBA68	TY-YFP-KKT8 tagging vector, Hygromycin (Akiyoshi and Gull, 2014)
pBA71	TY-YFP-KKT4 tagging vector, Hygromycin (Akiyoshi and Gull, 2014)
pBA72	TY-YFP-KKT7 tagging vector, Hygromycin (Akiyoshi and Gull, 2014)
pBA74	TY-YFP-KKT10 tagging vector, Hygromycin (Akiyoshi and Gull, 2014)
pBA96	TY-YFP-KKT16 tagging vector, Hygromycin (Akiyoshi and Gull, 2014)

Biology Open • Supplementary information

pBA97	TY-YFP-KKT14 tagging vector, Hygromycin (Akiyoshi and Gull, 2014)
pBA106	3FLAG-6HIS-YFP tagging vector, Hygromycin (this study)
pBA148	TY-tdTomato tagging vector, Blasticidin (Akiyoshi and Gull, 2014)
pBA164	TY-tdTomato-KKT2 tagging vector, Blasticidin (Nerusheva and Akiyoshi, 2016)
pBA310	Inducible expression vector, integrate at 177 bp, Phleomycin (Nerusheva and Akiyoshi, 2016)
pBA586	3FLAG-6HIS-YFP-CYC6 tagging vector, Hygromycin (this study)
pBA670	3FLAG-6HIS-YFP-CRK3 tagging vector, Hygromycin (this study)
pBA734	p2T7-177, CYC6 RNAi, integrate at 177 bp, Phleomycin (this study)
pBA1319	Inducible GFP-NLS-CYC6$^{\Delta 1-57}$ expression vector, integrate at 177 bp, Phlelomycin (this study)

Table S3. Primers and synthetic DNA sequences used in this study.

To make	Primer (all are listed 5' to 3') or synthetic DNA sequences
pBA31	MAP103 coding sequence (CDS) targeting sequence with *Xba*I and *Not*I<br>BA140: gatcgatc TCTAGA GGAGCAGGT TCCAAGGAGGCTCCACATCG<br>BA141: gatcgatc GCGGCCGC ACAAGATGAGAAGCCCTTTC<br>MAP103 5'UTR targeting sequence with *Not*I and *Bam*HI<br>BA142: gatcgatc GCGGCCGC GAAATATTGGTCTTTAAGTC<br>BA143: gatcgatc GGATCC AACCGCTACAGCTATAGTAA
pBA106	3FLAG-6HIS tag with *Hind*III and *Spe*I cut sequences<br>BA403: AGCTT<br>ATGGATTACAAGGATGACGACGATAAGGATTACAAGGATGACGACGAT<br>AAGGATTACAAGGATGACGACGATAAG CACCATCACCATCACCAT A<br>BA404: CTAGT<br>ATGGTGATGGTGATGGTGCTTATCGTCGTCATCCTTGTAATCCTTATCGT<br>CGTCATCCTTGTAATCCTTATCGTCGTCATCCTTGTAATCCAT A
pBA586	CYC6 CDS targeting sequence with *Xba*I and *Not*I<br>BA977: gatcgatc TCTAGA GGAGCAGGT AATCCCACGGCACTTCGTGA<br>BA978: gatcgatc GCGGCCGC ATACCGGATTATTCTCACGA<br>CYC6 5'UTR targeting sequence with *Not*I and *Bam*HI<br>BA979: gatcgatc GCGGCCGC CATTAGTTGAACGTCTAACG<br>BA980: gatcgatc GGATCC TGCCGTGCAGGACCCCTAAT
pBA670	Synthetic DNA for the N-terminal tagging target sequence for CRK3 with *Xba*I and *Bam*HI<br>TCTAGA GGAGCAGGT<br>acaatgcttggggcgttaaccggtcgacaactttcctctggtcttaaggatcagttcgaccgctataatcgaatggacatac<br>ttggagaaggaacgtatggagttgtgtaccgtgctgttgacagggcaacgggacagatcgtcgcactgaagaaagtga

Biology Open • Supplementary information

gattagatcgcaccgatgagggaatacctcaaacagctcttcgggaggtatctattttgcaagaaatccatcaccccaaca ttgttaGCGGCCGCtctgtatttagctatgatctcactatttccctcttttcttcgttgggttgtgtacctagcgatgttttac ctcgacaattctcggtcgaggggggctaaggcggtttctacccctatcaatctttgaaagaagttatgggtgtctcctcctac cttcatttattgcaaggtggttgctacataaaatttttatttttgtctctgctttcctcttaagttcttcaggaaacgttaggttgaa ggaggagat GGATCC

pBA734 CYC6 coding sequence (378 – 801 bp) with *Spe*I and *Hind*III
BA1249: gatcgatc ACTAGT AATGATTCTCGTCGATTGGC
BA1250: actgactg AAGCTT CTGCGATTGGATCTGCTGTA

pBA1319 CYC6 coding sequence (172–1278 bp) with *Pac*I and *Asc*I
BA1825: gatc TTAATTAA G TACAGCCCCGTAGGTACGGC
BA1828: gatc GGCGCGCC CTA AAAGTCAGGTACTTCACTAG

Supplemental references

Akiyoshi, B. and Gull, K. (2014). Discovery of unconventional kinetochores in kinetoplastids. *Cell* **156**, 1247–1258.

Hammarton, T. C., Clark, J., Douglas, F., Boshart, M. and Mottram, J. C. (2003). Stage-specific differences in cell cycle control in *Trypanosoma brucei* revealed by RNA interference of a mitotic cyclin. *J. Biol. Chem.* **278**, 22877–22886.

Katoh, K. and Standley, D. M. (2013). MAFFT multiple sequence alignment software version 7: improvements in performance and usability. *Mol. Biol. Evol.* **30**, 772–780.

Kelly, S., Reed, J., Kramer, S., Ellis, L., Webb, H., Sunter, J., Salje, J., Marinsek, N., Gull, K., Wickstead, B., et al. (2007). Functional genomics in *Trypanosoma brucei*: a collection of vectors for the expression of tagged proteins from endogenous and ectopic gene loci. *Mol. Biochem. Parasitol.* **154**, 103–109.

Li, Z. and Wang, C. C. (2003). A PHO80-like cyclin and a B-type cyclin control the cell cycle of the procyclic form of *Trypanosoma brucei*. *J. Biol. Chem.* **278**, 20652–20658.

Nerusheva, O. O. and Akiyoshi, B. (2016). Divergent polo box domains underpin the unique kinetoplastid kinetochore. *Open Biol* **6**, 150206.

Waterhouse, A. M., Procter, J. B., Martin, D. M. A., Clamp, M. and Barton, G. J. (2009). Jalview Version 2--a multiple sequence alignment editor and analysis workbench. *Bioinformatics* **25**, 1189–1191.

Wickstead, B., Ersfeld, K. and Gull, K. (2002). Targeting of a tetracycline-inducible expression system to the transcriptionally silent minichromosomes of *Trypanosoma brucei*. *Mol. Biochem. Parasitol.* **125**, 211–216.
